# Effect of cancer waiting time standards in the English National Health Service: a threshold analysis

**DOI:** 10.1186/s12913-024-11350-z

**Published:** 2024-08-14

**Authors:** Laura Quinn, Paul Bird, Richard Lilford

**Affiliations:** 1 Institute of Applied Health Research, University of Birmingham, Birmingham, UK; 2 NIHR Birmingham Biomedical Research Centre, University Hospitals Birmingham NHS Foundation Trust and University of Birmingham, Birmingham, UK; 3 Institute for Translational Medicine, University Hospitals Birmingham NHS Foundation Trust, Birmingham, UK; 4 Health Innovation West Midlands, Birmingham, UK

**Keywords:** Waiting time standards, Cancer care, Threshold analysis

## Abstract

**Background:**

The English National Health Service has multiple waiting time standards relating to cancer diagnosis and treatment. Targets can have unintended effects, such as prioritisation based on targets instead of clinical need. In this case, a `threshold effect’ will appear as a spike in hospitals just meeting the target.

**Methods:**

We conducted a retrospective study of publicly available cancer waiting time data, including a 2-week wait for a specialist appointment, a 31-day decision to first treatment and a 62-day referral to treatment standard that attracted a financial penalty. We examined the performance of hospital trusts against these targets by financial year to look for threshold effects, using Cattaneo et al. manipulation density test.

**Results:**

Trust performance against cancer waiting targets declined over time, and this trend accelerated since the start of the Covid-19 pandemic. Statistical evidence of a threshold effect for the 2-week and 31-day standard was only present in a few years. However, there was strong statistical evidence of a threshold effect for the 62-day standard across all financial years (*p* < 0.01).

**Conclusion:**

The data suggests that the effect of threshold targets alters hospital behaviour at target levels but does not do so equally for all standards. Evidence of threshold effects for the 62-day standard was particularly strong, possibly due to some combination of a smaller volume of eligible patients, a larger penalty, multiple waypoints where hospitals can intervene, baseline performance against the target and where the target is set (i.e. how much headroom is available). RCTs of the use of threshold targets and of different designs for such targets in the future would be extremely informative.

**Supplementary Information:**

The online version contains supplementary material available at 10.1186/s12913-024-11350-z.

## Background

The English National Health Service (NHS) provides an example of one European country with performance waiting time standards for diagnosing and treating suspected cancer patients. There are broadly two kinds of standards – one for people with suspected cancer and one for people who have confirmed cancer. The standards were introduced in the NHS Cancer Plan which was published 2000 and implemented in 2005 [1].

The first type of standard, for people with suspected cancer, arises when a patient is either referred to see a hospital doctor (specialist) for symptoms suspicious of cancer by their general practitioner (GP) or through a screening programme (breast cancer, bowel cancer or cervical screening) [2]. There are two standards of this type, both of which apply to patients referred by a GP or through a screening programme. The first of these is the 2-week wait (2WW) standard which stipulates that patients should be seen by a specialist within two weeks of referral. The second standard is the 28-day faster diagnosis standard (FDS), which stipulates that patients receive a diagnosis (positive or negative for cancer) within 28 days of referral. The 28-day FDS will replace the 2-week wait standard but since it was only introduced in 2021, it is not considered here (*see methods*) [3]. Over 90% of referred patients do not have a cancer diagnosis and when this is confirmed, they transfer to the 18-week referral to treatment standard that applies to all NHS patients in England [2].

The second type of standard applies to patients who do receive a cancer diagnosis. Again, these patients are covered by two standards. First, the 31-day decision to first treatment standard (DTT) applies to the length of time between a treatment decision being made and treatment starting. Unlike the other standards, the 31-day DTT standard applies to both patients with a new primary cancer and recurrent cancer. In addition to an overall 31-day target covering the initial treatment, whatever the modality, there are additional targets specific for the modality of any subsequent treatments (e.g. surgery for a patient who first had a drug treatment). The second standard, the 62-day referral to treatment standard (RTT), encompasses the length of time between referral and the treatment for cancer beginning. This standard encompasses the other standards (except for standards regarding subsequent treatments) and applies only to patients who receive a new primary diagnosis of cancer.

Many European countries have performance targets related to diagnosis or treatment times [4–6]. However, a recent organisation of economic co-operation and development (OECD) document makes clear that targets can be used for many purposes [7], including financial penalties, setting national policy, informing patient choice and providing patients a right to change provider. For the English NHS cancer waiting standards, both financial and reputational incentives are used. The financial incentives have varied by standard and by year, and include both financial penalties for breaching the targets and additional funds for meeting the targets. The NHS standard contract which is published yearly describes the financial penalties by standard [8], however information on financial penalties prior to 2013/14 when the current financial penalties were introduced. Although data continued to be collected, financial penalties were suspended during the Covid-19 pandemic along with penalties from numerous other key national performance indicators.

The effect of threshold targets on hospital behaviour for cancer waiting time standards is unknown. One qualitative study [9] discussed the possible negative unintended consequences of performance standards. The health service personnel interviewed in this qualitative study speculated and provided anecdotal evidence that incentives would have unintended consequences. First, hospitals a `long way’ from reaching the target would not be incentivised by the threshold target as they would either have surpassed the target or were so far from achieving it that the effort involved trying to reach the target would not be repaid. Second, hospitals close to the threshold target may `game’ the system by prioritising patients close to the threshold target rather than on clinical need or, worse, manipulate data. This study was undertaken to explore the gaming hypothesis. This type of corporate behaviour would show up as a spike in the number of hospital trusts at the threshold target, referred to as a threshold effect.

While threshold effects have not been examined for cancer waiting time targets, two recent studies which have examined threshold effects in other health contexts. The first study showed a threshold effect for the 18-week referral to treatment standard across all referrals (not just cancer) [10]. The second study showed that there was a threshold effect for hospital staff vaccination rate targets, which tracked the target as it was changed over different financial years [11]. The aim of this study is to examine for threshold effects in cancer waiting time standard targets in NHS England hospital trusts by financial year.

## Methods

### Study design

This is a retrospective observational study including all NHS hospital trusts in England. We report our results in line with the Reporting of Studies Conducted using Observations Routinely collected Data [12], an extension of the Strengthening the Reporting of Observational Studies in Epidemiology standards.

### Data collection

**Fig. 1:**
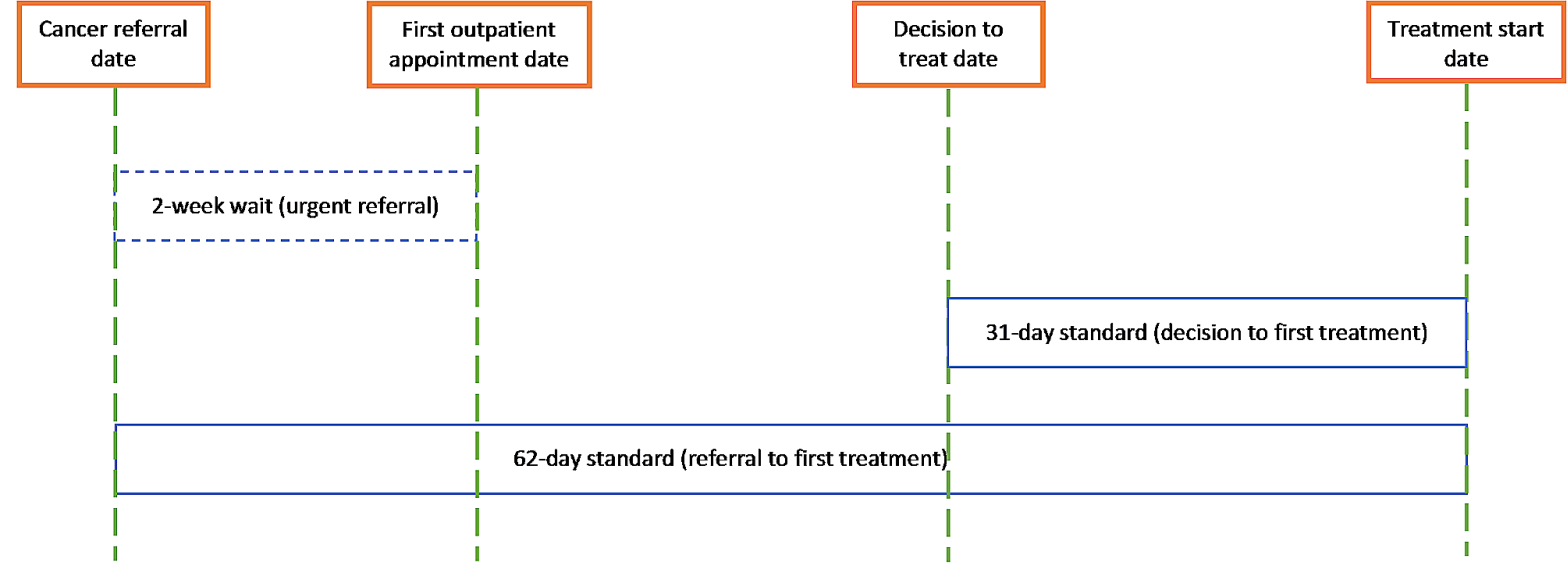
Description of NHS cancer waiting time standards. The 2-week wait standard applies to patients that may have cancer (dashed box), while the 31-day decision to first treatment standard and the 62-day referral to treatment standard apply to patients with a cancer diagnosis

Data were extracted on cancer waiting time standards for all hospital trusts from the NHS England website [https://www.england.nhs.uk/statistics/statistical-work-areas/cancer-waiting-times/] [13]. Data were included for 14 years (April 2010 to January 2023). We examined the 2-week wait (2WW), the 31-day decision to treatment (DTT) standards and the 62-day referral to treatment (RTT) standard. Descriptions are given in Table 1 and represented graphically in Fig. 1 [14]. As stated, the 28-day faster diagnosis standard (FDS) was introduced in 2021/22 and hospital trust performance measured against the standard only began in 2022/23, so the data for this standard was not included in the analysis.

At the end of each month, every NHS England hospital trust reports the total number of patients for each of the cancer waiting standards, showing the number of patients encompassed by the standard and the number of patients that breached the standard. From these values, hospital trust performance against the targets was calculated.

### Statistical methods

Monthly data was extracted for all NHS England hospital trusts on the cancer waiting target standards. We calculated the percentage of patients within the target in each hospital trust for each month. Then, we averaged the monthly data over financial years to show hospital performance by financial year.

We performed Cattaneo et al. manipulation density test [15] to check for evidence of a discontinuity at the target thresholds for each of the cancer waiting standards by financial year. For the 2-week wait standard, the target is 93%; for the 31-day decision to first treatment standard, the target is 96% and for the 62-day referral to treatment, the target is 85% [Table 1].

Cattaneo et al. test uses local polynomial regression models to estimate the density function of a running variable (percentage of patients treated within the target) and 95% confidence intervals either side of a specified target threshold. Bandwidth selection methods are used to select the optimal amount of data around the threshold target needed to check for evidence of discontinuity and only this data is used to produce the graphical representation of the results.

Local polynomial regression models used non-parametric techniques for estimating density functions, therefore they do not make strong parametric assumptions about its distribution and allow for modelling of curvature of non-linear patterns. All models were fit with quadratic polynomials (*p* = 2) for point estimates and cubic polynomials for confidence intervals (q = 3) therefore density estimates may lie outside the confidence intervals [16].

The null hypothesis for Cattaneo et al. test is that the density estimates at the target threshold are continuous. If the density estimates and 95% confidence intervals do not overlap or have little overlap at the threshold target (i.e. there is a spike in the density of hospital trusts at the target), we reject the null hypothesis showing there is evidence of discontinuity, which we refer to as a threshold effect. If the density estimates and 95% confidence intervals overlap at the threshold target, we accept the null hypothesis that the density is continuous around the threshold target and the evidence does not support a discontinuity or threshold effect. A further description of Cattaneo at al test is given in Supplementary material 1.

Cattaneo at al test was also carried out for the 31-day decision to subsequent treatment by type of treatment (radiotherapy, drug treatment and surgery).

All analysis were conducted used the rddensity package in Stata v18.0.

**Table 1:** Description of NHS cancer waiting time standards and threshold targets used in this study

Name of standard	Acronym	Description	Threshold target*	Financial penalty**
2-week wait standard	2WW	Patients should be seen by a **cancer specialist** within two weeks of an urgent referral by general practitioner **(GP) referral** or **screening programme** referral for suspected cancer	93%	£200
31-day decision to treatment standard	DTT	Patients should have their *first definitive treatment* of cancer within 31 days of having a **decision to treat^**	96%	£1,000
		Patients should have their *subsequent ***drug treatment** for cancer within 31 days	98%	
		Patients should have their *subsequent ***surgery** for cancer within 31 days	94%	
		Patients should have their *subsequent ***radiotherapy** for cancer within 31 days	94%	
62-day referral to treatment standard	RTT	Patients should have their **first definitive treatment** within 62 days of **referral**	85%	£1,000
28-day faster diagnosis standard^+^	FDS	Patients should not wait more than 28 days from urgent **referral** to having a **diagnosis of cancer or having cancer ruled out**	75%	-

*This refers to the percentage of patients who must meet the threshold target

**Financial penalty per patient from 2013/14 to 2020/21 reported in NHS England contracts

^+^The 28-day faster diagnosis standard (FDS) was not included in analysis as only introduced in 2021/22

^This threshold refers to first treatment given irrespective of the mix of modalities the patient eventually receives

## Results

**Fig. 2:**
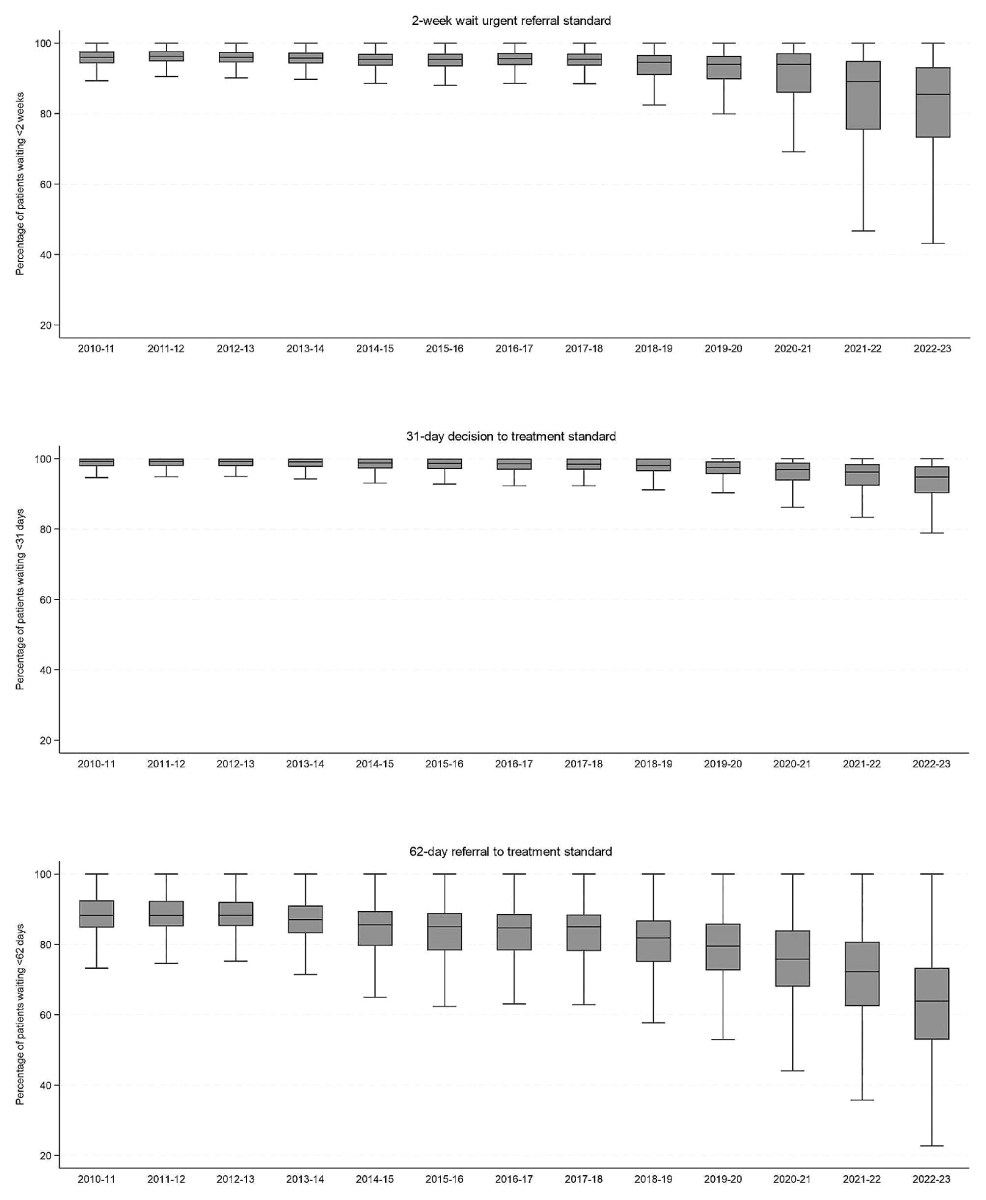
Box plot of percentage of patients waiting for each cancer waiting standard (2-week wait urgent referral, 31-day decision to treatment, and 62-day referral to treatment) across trusts from 2010/11 to 2022/23. Outliers were excluded

The number of NHS England hospital trusts ranged from 151 to 180 across financial years due to organisation changes (such as mergers and de-mergers) or hospital trusts not submitting their data for the standards [see Supplementary Table 1* for further details*]. The number of trusts meeting the 2-week wait for urgent referral, 31-day decision to first treatment and 62-day referral to treatment standard all declined over time [Fig. 2] and has not been met at aggregated national level since 2015 [17]. The decline precedes the Covid-19 pandemic, but the trend accelerates after 2020/21 and shows little or no sign of improvement in the post-Covid-19 epoch.

### 2-week wait urgent referral

**Fig. 3:**
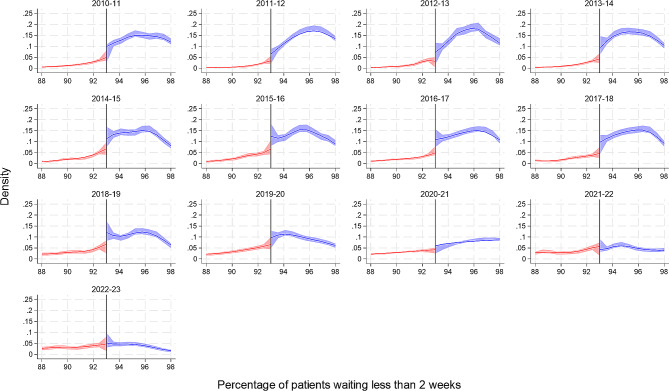
Cattaneo et al. manipulation density test for the 2-week wait urgent referral standard at the 93% target by financial year. The figure includes a curve which represents the local polynomial density estimates either side of the target threshold, and the shaded area represents the 95% confidence intervals

In 2010/11, on average (across all hospital trusts for all months), 96% (95% CI: 94 to 98) of patients were seen by a specialist within two weeks of an urgent GP referral, exceeding the 93% target. This reduced over time to a low of 85% (95% CI: 73 to 93) of patients in 2022/23. From Fig. 3, we observe visual evidence of a threshold effect, with a spike in the number of hospital trusts at the threshold target in most financial years prior to the start of the Covid-19 pandemic in 2020/21. However, this evidence is only statistically significant in three financial years: 2013/14, 2016/17 and 2018/19 [Table 2].

### 31-day decision to treatment standards

**Fig. 4:**
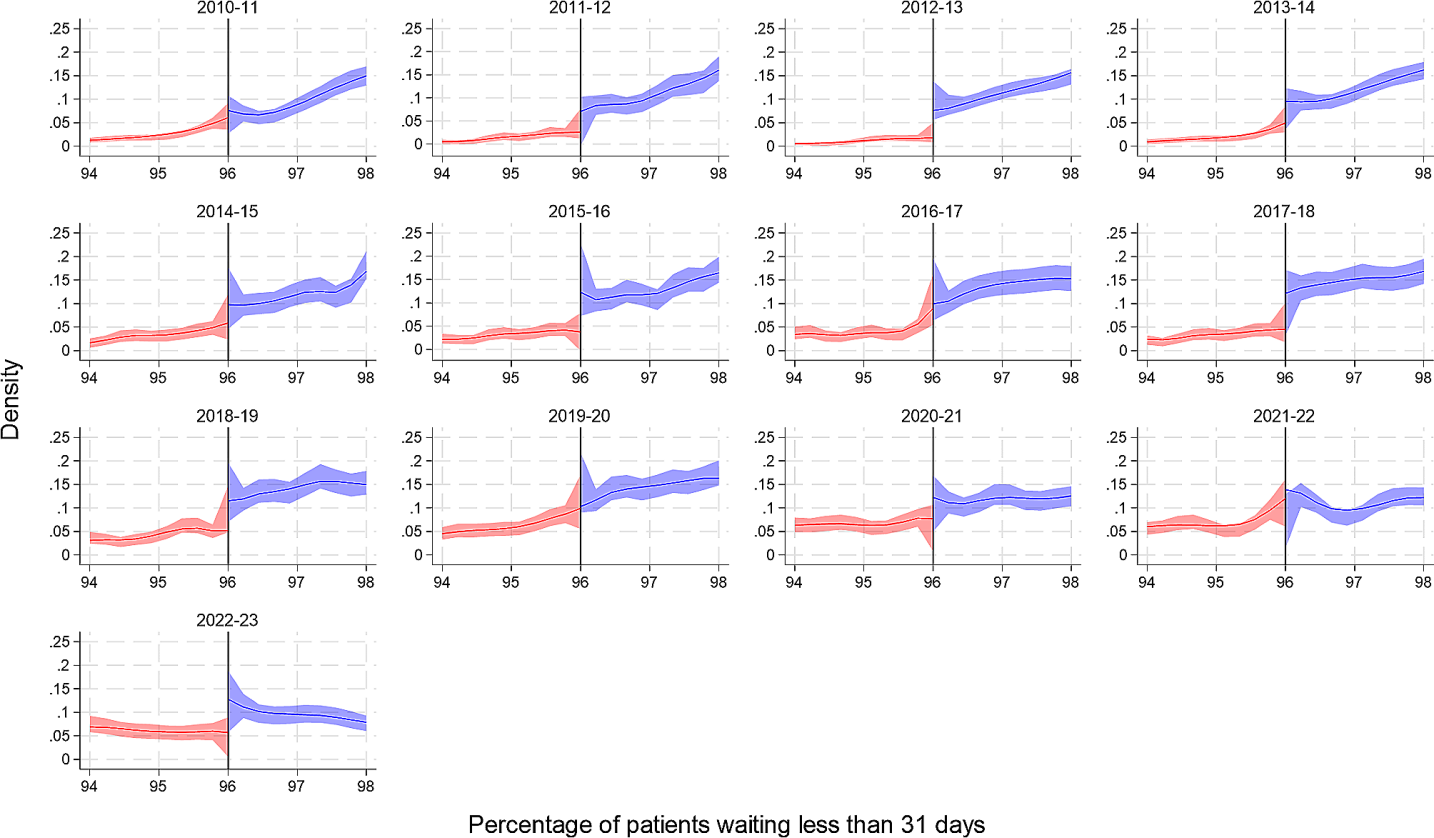
Cattaneo et al. manipulation density test for the 31-day decision to first treatment standard at the 96% target by financial year. The figure includes a curve which represents the local polynomial density estimates either side of the target threshold, and the shaded area represents the 95% confidence intervals

From 2010/11 to 2016/17, on average hospital trusts treated 99% of patients within 31 days of a decision to treat. This reduced to 98% for three years and only went below the 96% target to 95% (95% CI: 90–98%) in 2022/23. From Fig. 4, we again see visual evidence of a threshold effect for some financial years, however this was statistically significant in only three financial years (2012/13, 2015/16 and 2022/23). The results were similar for subsequent treatments when split by modality [see Supplementary Table 2].

### 62-day referral to treatment standard

**Fig. 5:**
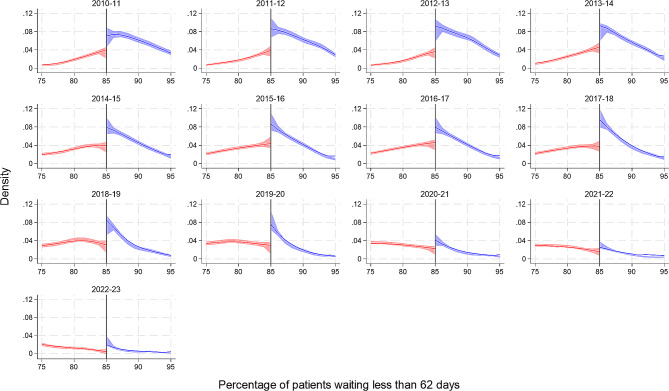
Cattaneo et al. manipulation density test for the 62-day referral to treatment standard at the 85% target by financial year. The figure includes a curve which represents the local polynomial density estimates either side of the target threshold, and the shaded area represents the 95% confidence intervals

The average percentage of patients treated within 62 days of being referred reduced from a high of 88% (95% CI: 85 to 93) in 2010/11 to a low of 64% (95% CI: 53 to 73) in 2022/23. For the 62-day referral to treatment standard, we can see the density estimate increases as it reaches the threshold target, followed by a large spike at the target which drops off afterwards. The increase before the threshold target disappears in later years after 2018/19, and the spike at threshold target also lessens, especially since the Covid-19 pandemic. There was both visual evidence and statistical evidence of a threshold effect across all financial years [Fig. 5; Table 2].

**Table 2:** Percentage of patients waiting within target for cancer waiting standards and p-values for Cattaneo et al. manipulation density test by financial year. Standards include 93% of patients waiting less than 2 weeks for first outpatient appointment since referral (2WW); 96% of patients waiting less than 31 days for first treatment since the decision to first treatment (31-day DTT) and 85% of patients waiting less than 62 days for treatment since referral (62-day RTT)

Financial year	2WW standard (Target 93%)		31-day DTT standard (Target 96%)		62-day RTT standard (Target 85%)	
	Median (IQR)	Robust *p*-value	Median (IQR)	Robust *p*-value	Median (IQR)	Robust *p*-value
2010/11	96 (94 to 98)	0.743	99 (98 to 100)	0.750	88 (85 to 93)	0.014*
2011/12	96 (95 to 98)	0.427	99 (98 to 100)	0.715	88 (85 to 92)	< 0.001*
2012/13	96 (95 to 97)	0.166	99 (98 to 100)	0.001*	88 (85 to 92)	< 0.001*
2013/14	96 (94 to 97)	0.022*	99 (98 to 100)	0.248	87 (83 to 91)	0.010*
2014/15	95 (94 to 97)	0.080	99 (97 to 100)	0.240	86 (80 to 89)	< 0.001*
2015/16	95 (93 to 97)	0.060	99 (97 to 100)	0.011*	85 (78 to 89)	< 0.001*
2016/17	96 (94 to 97)	0.015*	99 (97 to 100)	0.472	85 (78 to 89)	< 0.001*
2017/18	95 (94 to 97)	0.176	98 (97 to 100)	0.246	85 (78 to 88)	< 0.001*
2018/19	95 (91 to 97)	0.001*	98 (97 to 100)	0.190	82 (75 to 87)	< 0.001*
2019/20	94 (90 to 96)	0.486	98 (96 to 100)	0.078	80 (73 to 86)	< 0.001*
2020/21	94 (86 to 97)	0.630	97 (94 to 99)	0.556	76 (68 to 84)	0.005*
2021/22	89 (75 to 95)	0.427	96 (92 to 98)	0.263	72 (62 to 81)	0.016*
2022/23	85 (73 to 93)	0.493	95 (90 to 98)	0.048*	64 (53 to 73)	< 0.001*

* p-value < 0.05

## Discussion

### Summary of study

Three performance standards regarding the waiting times for the diagnosis and treatment of cancer patients in NHS England are examined in this study. The number of trusts meeting the 2-week wait urgent referral, 31-day decision to first treatment and 62-day referral to treatment standard has declined over time. For the 2-week wait, there was visual evidence of a threshold effect in all years up to the start of the Covid-19 pandemic, however this reached the conventional threshold for statistical significance in only three years. Likewise, for the 31-day decision to first treatment standard, visual evidence was corroborated by statistical evidence in only three years (and these were not the same three years as observed for the 2-week wait standard). For the 62-day referral to treatment, however there was both visual and statistical evidence of a threshold effect across all financial years. For the 62-day referral to treatment standard, we can see that the density estimate increases as it gets closer to the threshold target, then there is large increase or spike at the target which drops off after the 85% target. This spike at the 85% target is visually less impressive in later years, especially since the Covid-19 pandemic, however the effect remains significant at the 5% level.

### Comparison with literature

Although there are many healthcare performance standards, there are few studies looking at the effects of these targets and even less which carried out a formal threshold analysis. We have reviewed the literature on thresholds and to our knowledge this is the first study of the effect of threshold targets for cancer waiting time standards.

Previous studies looking at threshold effects include a study looking at targets for hospital staff vaccination rates, which showed effects tracking the threshold as the government changed it over different financial years [11]. Another study looked at the effect of the 18-week referral to treatment standard, which showed a threshold effect across all financial years at the 92% target until the Covid-19 pandemic when the majority of hospital failed to meet the target [10].

Our study provides a more mixed picture. There was only statistical evidence of a threshold effect for a few years for the 2-week wait and 31-day decision to first treatment standard while statistical evidence of threshold effect was present across all financial years for the 62-day referral to treatment standard which persisted even during the Covid-19 pandemic. This evidence therefore shows that threshold effects are not inevitable when a target is introduced and suggests that there are many factors which combine to produce threshold behaviour.

### Theoretical considerations

It is noteworthy that the 62-day referral to treatment standard for which the threshold effect is most obvious and statistically significant differs from the other standards in a few ways. These differences include the number of patients the standard applied to, the financial penalties imposed, the number of waypoints in the patient pathway, baseline hospital performance against targets and the amount of headroom for improvement beyond the threshold. In comparison to the 2-week standard, both the 31-day and 62-day standards trigger a higher financial penalty, and they apply to a smaller number of patients (as they only apply to patients with a new diagnosis of primary cancer) [Table 1]. However, there are further differences when comparing the 62-day standard to the other standards. The 62-day standard consolidates the other standards and includes the time between the first outpatient appointment and the decision to treat [Fig. 1] meaning there are multiple waypoints in the patient pathway where hospitals can intervene to meet the standard. The 62-day standard has a lower target at 85% (compared to 93% or 96%) meaning there is more headroom for improvement [Table 1] and worse baseline hospital performance compared to the other standards [Table 2] meaning the target is harder to meet. We hypothesise some combination of these differences could be the reason there is strong evidence of threshold effects for the 62-day standard and not for the other standards.

### Implications

The threshold effect shows that hospitals react to the headline threshold to which the financial penalty applied. Does this matter? We present two polar views and a compromise view. The first polar view would be that the threshold effect is of no consequence; the presence of the threshold effect does not exclude a general improvement as intended. The opposing polar view would draw attention to three potential negative effects. First, focus on the threshold implies that, to some extent at least, effort that could be spent on general improvement is deflected to manipulating waiting list to achieve the target. Second, the threshold effect suggests that proximity to the threshold, rather than clinical need, determines priority for treatment. Third, in-so-far as targets motivate hospitals near the threshold, they may de-motivate hospitals further from the threshold; a point suggested in the qualitative research cited in the Introduction. A compromise view would be that threshold effects contain important information for policy makers. First, they suggest caution in the use of targets. Second, policy makers should examine for threshold effects and perhaps try alternative designs such as reward adjusted against a sliding performance scale. And third, careful studies are required where threshold effects are compared head-to-head with no incentive or where one incentive design is compared to another. However, by demonstrating threshold effects, we raise the possibility that targets may do more harm than good. Hence the need for counterfactual studies.

The English Department of Health announced in August 2023 that it would retain the 31-day decision to first treatment and 62-day referral to treatment standards and replace the 2-week wait standard with the 28-day faster diagnosis standard [18]. This policy has been promulgated with no clear evidence of benefit. We provide some evidence that the policy may have negative side effects. Hence our plea for evidence-based policy in this area.

### Strengths and limitations

The main strength of this study is that the data on each of the cancer waiting standards for all hospital trusts in NHS England is publicly available on the NHS England website and is updated regularly. This study also used Cattaneo et al. manipulation density test, which is a recently developed test to check for evidence of discontinuity at threshold targets which requires little pre-specification. The main disadvantage of this study is that it is purely quantitative and any behavioural mechanisms or motivations that might explain why threshold effects are present for some standards and must be speculative. Nevertheless, we can draw on a previous behavioural literature to aid these speculations. We are unable to determine any variation in the threshold effect resulting from changes to the financial penalties prior to 2013/14 when current financial penalties were introduced as these are contained within local and national contracting documents which are not routinely available in the way performance data are. Financial penalties were also suspended during the Covid-19 pandemic but due to the whole scale disruption caused to delivery of health services in this period it is not possible to attribute any change to the suspension of financial penalties. Lastly, we do not directly observe the counterfactual state; a point to which we now turn.

## Conclusion

Targets can produce threshold effects even in the critically important topic of cancer care. However, threshold effects vary according to the incentive to reach the target. Threshold analysis provides an inexpensive method that would allow policymakers to gain some insight into the behavioural effects of their policies. The challenge for policymakers is to design standards that can improve performance without producing adverse effects. It would be extremely informative to conduct RCTs of the use of threshold targets and of different designs for such targets.

### Electronic supplementary material

Below is the link to the electronic supplementary material.

Supplementary Material 1

## Data Availability

This study used publicly available data which can be downloaded from: https://www.england.nhs.uk/statistics/statistical-work-areas/cancer-waiting-times/.

## Abbreviations

NHS: National health service
GP: General practitioner
2WW: 2 week wait
FDS: Faster diagnosis standard
DTT: Decision to treatment
RTT: Referral to treatment
OECD: Organisation of economic co–operation and development
