# Supplementary material for: Effect of cancer waiting time standards in the English National Health Service: a threshold analysis
Source: BMC Health Serv Res. 2024 Aug 14;24:929. doi: 10.1186/s12913-024-11350-z (PMC11323589; doi:10.1186/s12913-024-11350-z)
Supplement: Supplementary file 1 — Supplementary Material 1 [file 12913_2024_11350_MOESM1_ESM.docx]

## Supplementary material 1 – Description of Cattaneo et al manipulation density test

The Cattaneo et al. manipulation density test is a statistical method used to detect if there is evidence of a spike or discontinuity around a specific threshold target. This situation might happen when there is a reward based on whether a person or organisation meets or fails to meet their threshold target.

**Example: Cancer waiting time targets**

For example, let’s look at the 62-day referral to treatment standard for cancer waiting times. This target requires that, at the end of the month, 85% of patients in each NHS hospital should wait less than 62 days for their cancer treatment from the time they were referred by their doctor on suspicion of having cancer.

- A hospital meets the target if 85% of patients or more of its patients waited less than 62 days for their treatment.
- However, a hospital fails to meet the target if less than 85% of patients waited less than 62 days (i.e. more than 15% of patients waited over 62 days for treatment).

In an ideal situation, we would expect that the distribution of hospitals around the 85% was continuous, with no spikes or discontinuities. However, if hospitals manipulate their reported waiting times to fall just above the 85% cutoff (to avoid the penalties associated with failing to meet the target), we will see a spike or discontinuity at the 85% threshold target.

**Test methodology**

The Cattaneo et al. test looks for this kind of discontinuity or spike in the data distribution around the target threshold by performing the following steps:

1. **Estimating** **densities:** The test estimates the density (concentration) of hospitals hospital either side of the 85% threshold target using statistical models, which are called local polynomial regression models. Densities show how many hospital trusts are treating a specific percentage of patients with 62 days of referral.
2. **Checking for overlap:** The test checks if the estimated densities and their confidence intervals smoothly overlap at the 85% threshold target, or if there is a clear spike or discontinuity at 85%.
3. **Evidence of discontinuity:** If the confidence intervals for the densities do not overlap, or only overlap slightly at the threshold target, then evidence of discontinuity is detected. This would appear as a spike or discontinuity in the density at the 85% threshold, as shown in *Figure 1*.
4. **No evidence of discontinuity:** However, if the densities smoothly continue across the threshold target, with the confidence intervals clearly overlapping, it indicates no evidence of discontinuity at the threshold target, as shown in *Figure 2*.

The Cattaneo et al. test helps show whether hospitals may strategically choose which patients to treat in order to meet the 85% target for the 62-day referral to treatment standard. This test is used in various situations to assess the potential effects of threshold targets.

**Figure 1.** Cattaneo et al manipulation density test with *evidence of discontinuity* at the 85% target for the 62-day referral to treatment standard. The figure includes a curve which represents the local polynomial density estimates either side of the target threshold, and the shaded area represents the 95% confidence intervals. There is *no overlap at the 85% target* suggesting evidence of discontinuity.

**Figure 2.** Cattaneo et al manipulation density test with *no evidence of discontinuity* at the 85% target for the 62-day referral to treatment standard. The figure includes a curve which represents the local polynomial density estimates either side of the target threshold, and the shaded area represents the 95% confidence intervals. There is *almost complete overlap* at the 85% target suggesting *no evidence of discontinuity*.

## Supplementary table 1 – Number of hospital trusts with data reported by standard and financial year.

|  | **Number of hospital trusts** | | |
| --- | --- | --- | --- |
| **Financial year** | **2WW standard** | **31-day DTT standard** | **62-day RTT standard** |
| **2010/11** | 180 | 180 | 193 |
| **2011/12** | 178 | 182 | 188 |
| **2012/13** | 178 | 179 | 189 |
| **2013/14** | 166 | 180 | 186 |
| **2014/15** | 167 | 182 | 184 |
| **2015/16** | 164 | 172 | 183 |
| **2016/17** | 162 | 163 | 169 |
| **2017/18** | 162 | 168 | 172 |
| **2018/19** | 161 | 165 | 164 |
| **2019/20** | 163 | 159 | 165 |
| **2020/21** | 159 | 156 | 157 |
| **2021/22** | 151 | 149 | 155 |
| **2022/23** | 145 | 151 | 151 |

## Supplementary table 2 - 31-day decision to subsequent treatment standard by modality of treatment

**Table 1.** Percentage of patients waiting less than 31 days for subsequent treatment since decision to treat by modality of treatment and p-values for Cattaneo’s et al manipulation test by financial year.

|  | **Drug treatment**  **(Target 98%)** | | **Surgery**  **(Target 94%)** | | **Radiotherapy**  **(Target 94%)** | |
| --- | --- | --- | --- | --- | --- | --- |
| **Financial year** | **Median (IQR)** | **Robust**  **p-value** | **Median (IQR)** | **Robust**  **p-value** | **Median (IQR)** | **Robust**  **p-value** |
| **2010/11** | 100 (100 to 100) | 0.350 | 100 (97 to 100) | 0.008 | 99 (96 to 100) | 0.468 |
| **2011/12** | 100 (100 to 100) | 0.859 | 100 (97 to 100) | 0.965 | 99 (98 to 100) | 0.380 |
| **2012/13** | 100 (100 to 100) | 0.367 | 100 (97 to 100) | 0.588 | 99 (97 to 100) | <0.001* |
| **2013/14** | 100 (100 to 100) | <0.001* | 100 (97 to 100) | 0.031* | 99 (98 to 100) | 0.422 |
| **2014/15** | 100 (100 to 100) | 0.014* | 100 (95 to 100) | 0.011* | 99 (97 to 100) | 0.808 |
| **2015/16** | 100 (100 to 100) | 0.044 | 100 (95 to 100) | <0.001* | 99 (97 to 100) | 0.253 |
| **2016/17** | 100 (100 to 100) | 0.341 | 100 (95 to 100) | 0.254 | 99 (97 to 100) | 0.937 |
| **2017/18** | 100 (100 to 100) | 0.983 | 100 (95 to 100) | 0.256 | 99 (97 to 100) | 0.172 |
| **2018/19** | 100 (100 to 100) | 0.149 | 100 (93 to 100) | 0.290 | 98 (96 to 100) | 0.507 |
| **2019/20** | 100 (100 to 100) | 0.693 | 97 (91 to 100) | 0.572 | 98 (95 to 100) | 0.489 |
| **2020/21** | 100 (99 to 100) | 0.755 | 95 (86 to 100) | 0.046* | 98 (96 to 100) | 0.988 |
| **2021/22** | 100 (99 to 100) | 0.467 | 93 (83 to 100) | 0.278 | 98 (95 to 100) | 0.270 |
| **2022/23** | 100 (98 to 100) | 0.344 | 89 (77 to 100) | 0.170 | 96 (87 to 99) | 0.498 |

* p-value <0.05
